# Impact of Neighborhood Disadvantage on Gastrointestinal Morbidity in Extremely Low Birthweight Infants

**DOI:** 10.7759/cureus.86856

**Published:** 2025-06-27

**Authors:** Samantha Shaffer, Katie A Huff, James Slaven, Seethal A Jacob, Jason Z Niehaus

**Affiliations:** 1 Pediatrics, Indiana University School of Medicine, Indianapolis, USA; 2 Neonatology, Indiana University School of Medicine, Indianapolis, USA; 3 Biostatistics and Health Data Science, Indiana University School of Medicine, Indianapolis, USA; 4 Pediatric Hematology/Oncology, Riley Hospital for Children, Indianapolis, USA; 5 Center for Pediatric and Adolescent Comparative Effectiveness Research, Indiana University School of Medicine, Indianapolis, USA

**Keywords:** extremely low birthweight infant, necrotizing enterocolitis, nicu, prematurity, social determinants of health

## Abstract

Introduction: Necrotizing enterocolitis (NEC) and spontaneous intestinal perforation (SIP) are major causes of morbidity and mortality in extremely low birth weight (ELBW) infants. Social determinants of health (SDH) are associated with disparate outcomes for neonates admitted to the neonatal intensive care unit (NICU). This study sought to identify correlations between NEC, SIP, and SDH.

Methods: Retrospective cohort study of infants born between 22 and 25 weeks of gestation between 2017 and 2023. Utilizing the area deprivation index (ADI) as a marker for social vulnerability.

Results: The study included 156 patients. Infants with NEC had a statistically significant median ADI score (indicating more disadvantage) compared to those without NEC. No Black infants in the least disadvantaged deciles were diagnosed with NEC.

Conclusion: Babies from more disadvantaged backgrounds are more likely to develop NEC. This is even more pronounced in Black infants. Further investigation could lead to a more tailored feeding protocol to promote health equity.

## Introduction

Necrotizing enterocolitis (NEC) and spontaneous intestinal perforation (SIP) are significant sources of morbidity and mortality among extremely low birth weight (ELBW) infants [1]. Approximately 10% of ELBW infants will develop NEC, and 10-50% of those infants will die from NEC-related complications [2]. This has significant long-term sequelae, including more than 25% requiring surgical intervention, many of whom go on to develop long-term dependence on parenteral nutrition [2]. Longitudinally, the incidence of neurodevelopmental impairment is also significantly higher in children who developed NEC as infants; it is about twice the incidence of children who did not develop NEC [3,4]. Infants who develop surgical NEC are at increased risk for poor growth throughout childhood [1].

SIP is less common, but 3% of ELBW infants will still develop this complication [2]. ELBW infants who develop SIP are more likely to be shorter and weigh less through adolescence than ELBW infants who do not develop SIP [1]. Prior studies have shown that infants with SIP have a higher incidence of intraventricular hemorrhage (IVH), chronic lung disease, and nosocomial infections [5].

While the mechanisms for NEC and SIP are not completely understood, intestinal integrity is a significant contributor to both. With NEC, disruption of gut epithelium by intraluminal bacteria triggers inflammation, and further breakdown of the epithelial barrier likely contributes [6]. This breakdown and inflammation disrupt blood flow to the gut, causing tissue injury and necrosis. The development of SIP is less clear, though it is known that ELBW infants have thin intestinal mucosa that is likely prone to perforation [2]. Unlike NEC, SIP is not related to enteral feeds [2].

Families of infants requiring neonatal intensive care unit (NICU) level care are more likely to have low socioeconomic status (SES) compared to families of infants not requiring NICU level care [7]. Social determinants of health (SDOH) are known to affect an infant’s risk of prematurity, with mothers living in areas with higher levels of social vulnerability more likely to deliver prematurely [8]. Additionally, prior studies have shown that there may be an association between intestinal inflammation and SDOH in NICU infants [9]. However, the relationship between SDOH and the development of NEC and SIP is unknown. This study aimed to identify associations between SDOH and the incidence of NEC and SIP in extremely preterm infants.

## Materials and methods

This study was considered exempt by the Institutional Review Board. We performed a retrospective cohort study of all live births between 22 and less than 26 weeks of gestation at a level IIIB high-risk delivery hospital, Riley Hospital for Children at Indiana University Health, IN, USA, from 2017 to 2023 using the electronic medical record (EMR). Data collection was limited to the first 30 days of the subjects’ lives based on prior reports of the average onset of NEC at 16.7 days of life [5]. We excluded infants who died before 48 hours of life, as NEC and SIP rarely occur within the first 48 hours of life, and these infants rarely receive enteral feeds. Infants born at 26 weeks of gestation or greater were also excluded since the incidences of NEC and SIP decrease with increasing gestational age.

The area deprivation index (ADI) was used as a proxy for SES [10,11]. The ADI is a composite measure of neighborhood disadvantage at the census block level, comprised of 17 variables related to four main domains: income/employment, education, housing, and household characteristics. At the state level, ADI is reported as deciles from one (least disadvantaged) to 10 (most disadvantaged). At the national level, ADI is reported as percentiles from one (least disadvantaged) to 100 (most disadvantaged). 

Data collected from the medical record included development of NEC and/or SIP, gestational age (GA), development of IVH, birthweight (BW), small for gestational age (SGA; BW less than the 10th percentile for GA) status, maternal SES (determined by ADI), race (Black, White, Asian, Other, Unknown participants), ethnicity (Hispanic, non-Hispanic, Unknown participants), payer status (private, public, uninsured), hours to first enteral feed, and mortality. We were limited to the races listed, including Unknown and Other participants, due to what was reported in the EMR. There were three categories for NEC: no NEC, medical NEC, or surgical NEC. SIP was classified as SIP or no SIP. For this study, all grading of IVH [12] was simply categorized as “IVH.” We also documented the diagnosis of patent ductus arteriosus (PDA) and treatment received, and the diagnosis of pulmonary hypertension (pHTN).

Basic descriptive demographic statistics were generated, giving continuous variables as means (standard deviations), medians (ranges), and categorical variables as frequencies (percentages). Bivariable analyses were then performed to determine if there were significant associations in either or combined NEC and SIP groups with demographics and with ADI groups, using Wilcoxon non-parametric tests for continuous outcomes, due to skewness, or chi-square tests for categorical outcomes, using Fisher’s Exact when >20% of cells had an expected count of <5. Due to possible differences between Black and White participants, analyses were also stratified by race. All analytic assumptions were verified. Analyses were performed using SAS v9.4 (SAS Institute, Cary, NC). A p-value <0.05 was used to define statistical significance.

## Results

From 2017 to 2023, 156 patients met the inclusion criteria and can be found in Table 1. Of the patients, 44.2% were male. The median GA was 24.7 weeks. Around 48.7% were White patients, 45.5% were Black patients, 3.8% were Asian patients, and 2% were Other or Unknown patients. Race was unable to be ascertained for one subject, as was ethnicity for a different subject; otherwise, all data points were complete for every subject. The median state ADI was 6.2, and the median national ADI was 73.9. For gastrointestinal-related morbidities, 12.8% developed surgical NEC, 9.0% developed medical NEC, and 16% developed SIP.

**Table 1:** Demographics ADI: area deprivation index; NEC: necrotizing enterocolitis; SGA: small for gestational age; SIP: spontaneous intestinal perforation. Data are presented as numbers (percent) unless noted, n=156. *Median (ranges).

Demographics	Data
Sex	
Female	87 (55.8)
Male	69 (44.2)
Race	
Asian patients	6 (3.8)
Black patients	71 (45.5)
White patients	76 (48.7)
Other patients	2 (1.3)
Unknown patients	1 (0.6)
Ethnicity	
Hispanic patients	25 (16.0)
Non-Hispanic patients	130 (83.3)
Unknown patients	1 (0.6)
*State ADI	7 (1 - 10)
*National ADI	80.5 (14 - 100)
*Gestational age (weeks)	24.7 (22.4 - 25.9)
*Birth weight (g)	673.5 (360 - 1006)
SGA	16 (10.3)
Died	45 (28.9)
SIP	25 (16.0)
NEC in the First Month	
No	122 (78.2)
Medical	14 (9.0)
Surgical	20 (12.8)

Table 2 shows the relationship between NEC and ADI. Patients with NEC (surgical or medical) had greater neighborhood disadvantage than patients without NEC, as indicated by median state (8 vs. 6, p=0.042) and national ADI (86.5 vs. 75, p=0.046). These two groups had similar incidences of maternal betamethasone administration, chorioamnionitis, preterm premature rupture of membranes (PPROM), IVH, pHTN, PDA, and hours to first feed. There was no significant difference in other demographic factors, including GA, sex, race, ethnicity, or payer. 

**Table 2:** Necrotizing enterocolitis ADI: area deprivation index; IVH: intraventricular hemorrhage; PDA: patent ductus arteriosus; PHTN: pulmonary hypertension; SGA: small for gestational age; SIP: spontaneous intestinal perforation; PPROM: preterm premature rupture of membranes; NEC: necrotizing enterocolitis; HUS: head ultrasound. Comparison of groups NEC versus no NEC, including ADI. Data are presented as numbers (percentages) unless noted. *Median (ranges).

Characteristics	No NEC	NEC (Medical or Surgical)	p-value
N	122	34	
Demographic Variables			
*Gestational age (weeks)	24.6 (22.4 – 25.9)	24.8 (22.9 – 25.9)	0.635
Sex			
Female	71 (58.2)	16 (47.1)	0.248
Male	51 (41.8)	18 (52.9)	
Race			
Asian patients	5 (4.1)	1 (2.9)	0.897
Black patients	53 (43.4)	18 (52.9)	
White patients	61 (50.0)	15 (44.1)	
Other patients	2 (1.6)	0 (0)	
Unknown patients	1 (0.8)	0 (0)	
Ethnicity			
Hispanic patients	21 (17.2)	4 (11.8)	0.217
Non-Hispanic patients	101 (82.8)	29 (85.3)	
Unknown patients	0 (0)	1 (2.9)	
*ADI State	6 (1 – 10)	8 (1 – 10)	0.042
*ADI National	75 (14 – 100)	86.5 (25 – 100)	0.046
Payer			
Public	90 (73.8)	31 (91.2)	0.097
Private	26 (21.3)	3 (8.8)	
Uninsured	6 (4.9)	0 (0)	
Maternal Variables			
Betamethasone (yes)	83 (68.6)	27 (79.4)	0.286
Chorioamnionitis (yes)	10 (8.3)	3 (8.3)	>0.999
PPROM (yes)	8 (6.6)	5 (14.7)	0.128
Neonate Outcomes			
*Birth weight (g)	670 (360 – 999)	699.5 (438 – 1006)	0.911
SGA (yes)	12 (9.8)	4 (11.8)	0.752
Died (yes)	31 (25.4)	14 (41.2)	0.073
IVH			
No	81 (6.4)	16 (47.1)	0.373
Grade 1	7 (5.7)	3 (8.8)	
Grade 2	13 (10.7)	5 (14.7)	
Grade 3	5 (4.1)	3 (8.8)	
Grade 4	10 (8.2)	5 (14.7)	
Cerebellar	3 (2.5)	1 (2.9)	
No HUS	3 (2.5)	1 (1.9)	
PHTN (yes)	24 (19.7)	8 (23.5)	0.858
PDA			
No	74 (60.7)	23 (67.7)	0.926
No Echo	22 (18.0)	6 (17.7)	
Yes (medical treatment)	17 (14.0)	4 (11.7)	
Yes (cath treatment)	9 (7.4)	1 (2.9)	

There was no difference in state or national ADI between infants with SIP and those without SIP, as shown in Table 3. There was also no difference in other demographic factors, including GA, sex, race, ethnicity, or payer. Infants with SIP were more likely to have a lower birth weight (p=0.031), develop IVH (p=0.006), or die (p=0.005). There was no difference between the two groups for maternal betamethasone use, chorioamnionitis, PPROM, pHTN, PDA, or hours to first feeds. 

**Table 3:** Spontaneous intestinal perforation ADI: area deprivation index; IVH: intraventricular hemorrhage; NEC: necrotizing enterocolitis; PDA: patent ductus arteriosus; PHTN: pulmonary hypertension; SGA: small for gestational age; PPROM: preterm premature rupture of membranes; HUS: head ultrasound. Data are presented as numbers (percentages) unless noted.

Characteristics	SIP=Yes	SIP=No	p-value
N	25	131	
Demographic Variables			
GA weeks	24.4 (22.9 – 25.9)	24.7 (22.4 – 25.9)	0.096
BW	606 (360 – 1006)	683 (386 – 999)	0.031
SGA (yes)	4 (16.0)	12 (7.2)	0.291
Sex			
Female	11 (44.0)	76 (58.0)	0.196
Male	14 (56.0)	55 (42.0)	
Race			
Asian patients	1 (4.0)	5 (3.8)	0.917
Black patients	13 (52.0)	58 (44.3)	
White patients	11 (44.0)	65 (49.6)	
Other patients	0 (0)	2 (1.5)	
Unknown patients	0 (0)	1 (0.8)	
Ethnicity			
Hispanic patients	5 (20.0)	20 (15.3)	0.115
Non-Hispanic patients	19 (76.0)	111 (84.7)	
Unknown patients	1 (4.0)	0 (0)	
ADI State	8 (1 – 10)	6 (1 – 10)	0.106
ADI National	86 (41 – 99)	75 (14 – 100)	0.133
Payer			
Public	22 (88.0)	99 (75.6)	0.492
Private	3 (12.0)	26 (19.9)	
Uninsured	0 (0)	6 (4.6)	
Maternal Variables			
Betamethasone (yes)	19 (76.0)	91 (70.0)	0.636
Chorioamnionitis (yes)	3 (12.0)	10 (7.8)	0.444
PPROM (yes)	2 (8.0)	11 (8.4)	>.999
Neonate Outcomes			
Died (yes)	13 (52.0)	32 (24.4)	0.005
IVH			
No	8 (32.0)	89 (67.9)	0.006
Grade 1	2 (8.0)	8 (6.1)	
Grade 2	5 (20.0)	13 (9.9)	
Grade 3	2 (8.0)	6 (4.6)	
Grade 4	4 (16.0)	11 (8.4)	
Cerebelar	2 (8.0)	2 (1.5)	
No HUS	2 (8.0)	2 (1.5)	
PHTN (yes)	5 (20.0)	27 (20.6)	0.353
PDA			
No	14 (56.0)	83 (63.4)	0.617
No echo	7 (28.0)	21 (16.0)	
Yes (medical treatment)	2 (8.0)	19 (14.6)	
Yes (cath treatment)	2 (8.0)	8 (6.1)	
Hours to feeds	34.4 (4.8 – 344.0)	27.6 (1.8 – 955.5)	0.270

A comparison of the top three deciles (less disadvantaged group) and bottom three deciles (more disadvantaged group) of ADI is shown in Table 4. There were 33 patients in the less disadvantaged group and 70 patients in the more disadvantaged group. There was no significant difference in the incidence of SIP (p=0.168) or NEC (p=0.053). Patients in the more disadvantaged group were more likely to receive public insurance than those in the less disadvantaged group (p<0.001). 

**Table 4:** Area deprivation index (ADI) NEC: necrotizing enterocolitis; SIP: spontaneous intestinal perforation. Comparison of the top three deciles and the bottom three deciles. Data are presented as numbers (percentages) unless noted.

Characteristics	ADI Top 3 (1, 2, 3)	ADI Bottom 3 (8, 9, 10)	p-value
All participants	N=33	N=70	
SIP			
Yes	3 (9.1)	15 (21.4)	0.168
No	30 (90.9)	55 (78.6)	
NEC			
Yes	4 (12.1)	21 (30.0)	0.053
No	29 (87.9)	49 (70.0)	
Payer			
Public	17 (51.5)	62 (88.6)	<0.001
Private	14 (42.4)	5 (7.1)	
Uninsured	2 (6.1)	3 (4.3)	
Black participants	N=9	N=30	
SIP			
Yes	0 (0)	10 (33.3)	0.079
No	9 (100)	20 (66.7)	
NEC			
Yes	0 (0)	13 (43.3)	0.018
No	9 (100)	17 (56.7)	
Payer			
Public	8 (88.9)	29 (96.7)	0.413
Private	0 (0)	0 (0)	
Uninsured	1 (11.1)	1 (3.3)	
White participants	N=21	N=37	
SIP			
Yes	3 (14.3)	4 (10.8)	0.699
No	18 (85.7)	33 (89.2)	
NEC			
Yes	4 (19.1)	7 (18.9)	>0.999
No	17 (81.0)	30 (81.1)	
Payer			
Public	8 (38.1)	30 (81.1)	<0.001
Private	13 (61.9)	5 (13.5)	
Uninsured	0 (0)	2 (5.4)	

Additionally, in Table 4, the more and less disadvantaged groups were divided by race. There were 21 White patients and nine Black patients in the less disadvantaged group, and 37 White patients and 30 Black patients in the more disadvantaged group. For White patients, there was no significant difference in the incidence of SIP or NEC (p=0.699 and p>0.999, respectively). Black patients in the less disadvantaged group were less likely to develop NEC than those in the more disadvantaged group (p=0.018). For Black patients, there was no significant difference between the two groups for the incidence of SIP (p=0.079). In fact, no Black patients in the less disadvantaged group were diagnosed with NEC or SIP. 

## Discussion

SDOH has been shown to have a significant impact on maternal and infant health [13]. This study describes the association between maternal socioeconomic neighborhood disadvantage, as measured by ADI, and the incidence of NEC and SIP, where a correlation was found between increasing neighborhood disadvantage and NEC. This correlation does not hold true with SIP, another cause of intestinal morbidity and mortality. Other groups have also reported an association between ADI and NICU outcomes, including NEC. However, while they noted an association between higher ADI (more disadvantaged) and the combined outcome of sepsis or NEC, there was no significant association between race and sepsis or NEC [14]. Of note, they also described an association between ADI and neonatal mortality and severe IVH, outcomes that we described when comparing the specific neonatal morbidities of NEC and SIP but did not analyze separately by ADI in our population.

The underlying cause of the correlation between ADI and NEC is unknown. A standardized feeding protocol within our institution, which includes almost ubiquitous breast milk or donor breast milk use, makes exclusive formula feeding in ELBW patients, a known risk factor for NEC, extremely rare [15]. Combining the application of a standardized feeding protocol with no difference in birth weight or GA, these groups are very similar in their major known risk factors for NEC. However, the differences in the nutritional components of maternal breast milk between the two groups are unknown. Perhaps the higher likelihood of food deserts or other barriers to obstetric care in the more disadvantaged mothers leads to a higher risk and thus incidence of NEC. Other studies have shown that there is a difference in breast milk composition between low- and high-income mothers [16]. This difference in composition may partly contribute to the increased incidence of NEC seen in the most disadvantaged cohort of Black infants compared to their least disadvantaged counterparts. 

Our study noted differences in the incidence of NEC when stratifying Black and White infants into the most disadvantaged and least disadvantaged ADI groups. There was essentially no difference in the incidence of NEC between the most and least disadvantaged White infants. SDOH does not seem to have a large effect on White infants. For White infants, approximately 19% of the most and least disadvantaged groups were diagnosed with NEC. However, this relationship was not true for Black infants. There is a statistically significant difference between the most and least disadvantaged groups amongst Black infants, with over 40% of the most disadvantaged Black infants diagnosed with NEC, while 0% of the least disadvantaged Black infants were diagnosed with NEC (or SIP). Further investigation is needed to understand how socioeconomic disparities may mediate racial disparities and the likelihood of NEC to address health inequities. While this is a small sample size, if this were to remain consistent across multiple centers, this could have major implications for designing an equitable, individualized feeding protocol.

This study does have limitations. The data included in the analysis for this study were limited by the information documented in the electronic medical record. In addition, the percentage of breast milk, donor breast milk, and formula was unknown for all of the patients due to limitations in the medical record. While our feeding protocol and high use of donor breast milk make it rare for formula use early on in the care for an ELBW infant, that information was unknown for this study. Additionally, the small sample size and single-center nature limit the generalizability of our results to different geographic locations with variable exposures and neighborhood advantages.

## Conclusions

This study found that maternal SDOH, as measured by ADI, is associated with NEC, though this was not seen with SIP. More specifically, Black infants in the least disadvantaged cohort were not diagnosed with NEC or SIP. This correlation was unique to Black infants, as there was no difference in NEC or SIP rates in the most and least disadvantaged White cohort. Understanding the drivers of this relationship is necessary to improve maternal and infant outcomes and requires a larger, multi-center study to better characterize the impact. 
